# Automation of *Caenorhabditis elegans* lifespan assay using a simplified domain synthetic image-based neural network training strategy

**DOI:** 10.1016/j.csbj.2023.10.007

**Published:** 2023-10-10

**Authors:** Antonio García-Garví, Pablo E. Layana-Castro, Joan Carles Puchalt, Antonio-José Sánchez-Salmerón

**Affiliations:** Instituto de Automática e Informática Industrial, Universitat Politècnica de València, Camino de Vera S/N, Valencia, 46022, Spain

**Keywords:** C. elegans, Lifespan automation, Deep learning, Training strategy, Synthetic data

## Abstract

Performing lifespan assays with *Caenorhabditis elegans* (*C. elegans*) nematodes manually is a time consuming and laborious task. Therefore, automation is necessary to increase productivity. In this paper, we propose a method to automate the counting of live *C. elegans* using deep learning. The survival curves of the experiment are obtained using a sequence formed by an image taken on each day of the assay. Solving this problem would require a very large labeled dataset; thus, to facilitate its generation, we propose a simplified image-based strategy. This simplification consists of transforming the real images of the nematodes in the Petri dish to a synthetic image, in which circular blobs are drawn on a constant background to mark the position of the *C. elegans*. To apply this simplification method, it is divided into two steps. First, a Faster R-CNN network detects the *C. elegans*, allowing its transformation into a synthetic image. Second, using the simplified image sequence as input, a regression neural network is in charge of predicting the count of live nematodes on each day of the experiment. In this way, the counting network was trained using a simple simulator, avoiding labeling a very large real dataset or developing a realistic simulator. Results showed that the differences between the curves obtained by the proposed method and the manual curves are not statistically significant for either short-lived N2 (p-value log rank test 0.45) or long-lived *daf-2* (p-value log rank test 0.83) strains.

## Highlights

• Proposal of a method using artificial neural networks to automate survival curve acquisition in *C. elegans* lifespan assays.
• Proposal and evaluation of a training method based on a simplified domain counting model with fully synthetic images.
• Neural network models for the detection of *C. elegans* have been compared with traditional image processing techniques.
• The models trained with synthetic data were tested with real lifespan assay data.

## Graphical abstract

**Figure:**
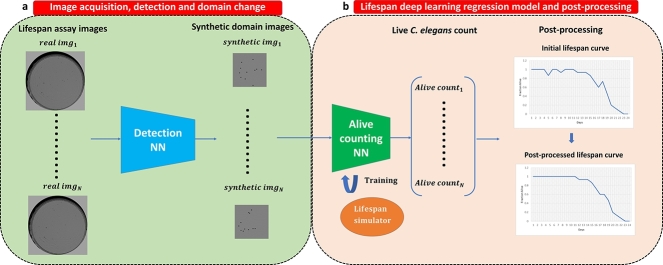

## 1 Introduction

There is great interest in understanding aging and this field is under continuous study. With aging, neurodegenerative diseases such as Alzheimer's and Parkinson's appear and constitute a major social problem. For this reason, it is essential to search for new drugs, therapeutic components and food products to help tackle these diseases and improve the quality of life. The nematode *Caenorhabditis elegans* (*C. elegans*) is an ideal animal model for research due to its characteristics: (a) it measures 1 mm in length, allowing large populations to be cultured in standard Petri dishes economically; (b) it is mainly fed on the bacteria *Escherichia coli* (*E.coli*), which is inexpensive; (c) it is transparent, making it possible to observe its tissues and organs under a microscope; (d) it has a short lifespan of approximately 3 weeks (although this can vary depending on the strain), allowing short-term assays to be performed. Thanks to research using this nematode, discoveries related to aging have been made [1], [2], [3]. The assay *par excellence* in the study of aging with *C. elegans* is the lifespan assay, which consists of counting the number of live nematodes each day of the experiment to obtain survival curves [4], [5], [6]. The nematodes are divided into different groups and subjected to different conditions that may influence life expectancy. Subsequently, statistical methods are used to analyze whether there are statistically significant differences between the survival curves obtained. This daily counting is carried out manually in most laboratories by qualified technicians, who check whether each nematode is still mobile by prodding them with a platinum wire to check their response, especially in the last days, when their movement is greatly reduced. Considering the above, together with the fact that 100 nematodes are usually needed to test each condition, this is a very laborious and time-consuming task. Thus, the automation of this process becomes highly necessary, as in addition to reducing time and facilitating the researcher's tasks, it provides more precise and objective measurements and avoids possible human errors. However, the automation of *C. elegans* assays in standard Petri dishes is a complex task as these nematodes can adopt a great variety of postures and give false positives (due to the presence of lint, dust, etc.) or false negatives (occlusions, aggregations, condensations). Solving these problems requires complex vision algorithms, in which numerous parameters must be manually adjusted. For this reason, given the good results obtained by deep learning techniques in task resolution in recent years, this work has sought to analyze the use of artificial neural networks to automate lifespan assays. All these problems make lifespan automation a task in which nematode detection and event counting (live or dead) must be solved. Training a neural network to learn to solve this problem requires complex models, which need very large labeled datasets in order to achieve good results. Obtaining very large datasets from lifespan experiments requires a lot of capture and labeling time. Developing a realistic simulator is also a complicated task and, furthermore, training these models require high computational resources. Due to these limitations of artificial neural networks when few data and computational resources are available, we propose a method that seeks to decompose the problem into two phases and to train the counting network with a simplified simulated image. In summary, the contributions of this paper are as follows:

• A method using artificial neural networks is proposed to automate the acquisition of survival curves for the lifespan assay with *C. elegans*. Specifically, it is approached as a regression problem in which the input of the network is a sequence with an image corresponding to each day of the experiment and the output is a sequence of values corresponding to the number of live nematodes each day.
• Given the difficulty of the problem, due to all the issues mentioned above together with the difficulty involved in obtaining a sufficiently large dataset, we propose a training method based on a simplified domain in order to train the regression network model with simulated images. The strategy consists of transforming the real images into a synthetic domain that is easy to simulate and, thus, train the regression network in this synthetic domain with as large a dataset as required.
• This strategy comprises two phases. First, a Faster R-CNN network is in charge of performing nematode detection, and the positions are used to obtain an image of simple blobs. Second, an alive counting neural network is used to perform the regression and obtain the lifespan curves.
• The proposed strategy could be applied to other animal behavior monitoring problems (trajectory analysis, anomaly detection) where obtaining large labeled datasets is costly.

The paper is structured as follows: Sect. 2 shows the state of the art in lifespan automation techniques with traditional computer vision and deep learning, and a review of techniques for training neural networks with synthetic data. Sect. 3 describes the proposed lifespan method. Experiments and results are presented in Sect. 4. Discussion and conclusions are presented in Sect. 5.

## 2 Related work

In this section we review the state of the art of automation lifespan experiments using both traditional computer vision and deep learning techniques and review some learning techniques using synthetic data.

### 2.1 Automation of *C. elegans* lifespan assays

In recent decades, different methods have been proposed to automate *C. elegans* lifespan assays [7]. WormScan [8] proposes the use of scanners to monitor standard Petri dishes and employ motion detection techniques to determine whether nematodes are alive or dead. The Lifespan Machine [9] also proposes a method for lifespan automation based on monitoring Petri dishes by means of scanners but in a more sophisticated way. Modifications have been made to the scanner allowing for better temperature regulation, which affects nematode lifespan, and optics are adjusted to obtain better image quality. Moreover, they developed their own software that enables them to identify the nematodes and analyze their movement to determine whether they are alive or dead. WorMotel [10] used non-standard multiwell plates, in which each nematode is separated, thus avoiding aggregations and facilitating the detection of dead nematodes. However, being in a very confined space limits the nematode's movement and may modify its behavior. Automated Wormscan [11] presents another method based on capture by scanners and has developed motion-based image analysis software. WormBot [12] is a robotic system that allows semi-automatic lifespan analysis. Recently, a new prototype [13] has been developed, capable of automating the lifespan assay and obtaining survival curves using traditional computer vision techniques. These methods automate nematode counting using traditional computer vision techniques based on motion analysis. The drawback of these techniques is that they require manual adjustment of numerous parameters that should work in order to solve a wide variety of problems arising in the assays: condensation, occlusions, aggregations, occurrence of fuzz and dust motes, changes in illumination, etc. Given these difficulties, it is interesting to analyze the use of artificial neural networks to solve lifespan assay automation, as they have recently demonstrated good results in object detection, classification and segmentation tasks [14], [15], [16], outperforming traditional techniques. In addition, neural networks have been used in recent years to solve problems [17], [18], [19], [20] related to people counting, behavioral analysis, etc. In this work we aim to count the number of live *C. elegans* on each day of the experiment. To know whether a nematode is alive or dead we must check if it has moved from one day to the next; therefore, this is an event counting and movement analysis problem. Several deep learning approaches have been proposed in the literature to solve different problems involving *C. elegans*. WorMachine [21] uses a neural network to distinguish nematodes from noise. In [22] convolutional neural networks (CNNs) are used to classify different strains of nematodes. We have also found papers proposing methods for the identification of head and tail, and the estimation of skeletons [23], [24], [25]. Works such as [26] and [27] use Faster R-CNN to detect *C. elegans*. Recently, methods based on neural networks working with microscopic images have been proposed: [28] outlines a method to segment and estimate the age of *C. elegans*; [29] uses linear regression and logistic regression models to estimate the age of nematodes; in [30] models are presented to classify nematodes into long-lived and short-lived categories, to classify movement into fast or slow and allow the accurate segmentation of nematodes into anterior, mid-body and posterior parts. In our previous work [31], we combined traditional vision techniques with a deep learning-based *C. elegans* live or dead classifier. Unlike our previous approach, where the problem was solved by individually classifying *C. elegans* as live or dead by combining traditional computer vision techniques and neural networks, in this work it has been approached as a regression problem based entirely on deep learning.

### 2.2 Synthetic datasets

One of the limitations of supervised learning with artificial neural networks is the difficulty in obtaining a sufficiently large, varied, and representative dataset of the problem under study. Depending on the problem, obtaining a correctly labeled dataset can be very laborious and costly. There are various techniques that can help to solve this problem, such as data augmentation by means of transformations (rotations, translations, intensity changes, etc.), the application of transfer learning or the use of synthetic imaging. The latter has stirred interest in the research community in recent years. The use of simulators can provide synthetic datasets, avoiding the cost of labeling and obtaining perfectly labeled images. However, the generated images usually present a problem known as domain transfer or domain gap, which prevents the direct use of the simulator-generated images to train models that work with real images. In recent years, different methodologies have been proposed to mitigate the domain gap problem [32]. One of these is domain randomization [33], which consists of applying transformations to the synthetic images so that the synthetic dataset distribution is sufficiently varied to make the model, trained with these data, robust enough to work with real data. Another technique is “cut, paste and learn” [34], which consists of mixing different real images to obtain synthetic samples. Despite reducing the domain gap, this technique is limited because the potential variability depends on the real images available. Recently, domain adaptation techniques [35] have been used to make the simulated images more realistic using generative adversarial networks (GANs). This refinement has also been performed in the reverse way, i.e., making real images pass into the domain of synthetic images. Other strategies employed are hybrid datasets [36]: (a) mixing real and simulated images; (b) first training the network with a simulated image and then performing the fine-tuning training with the real image. In this work, we propose a domain translation strategy from real to synthetic images, but we do not use a realistic synthetic image but, rather, a simplified image of blobs.

## 3 Proposed lifespan method

**Fig. 1:**
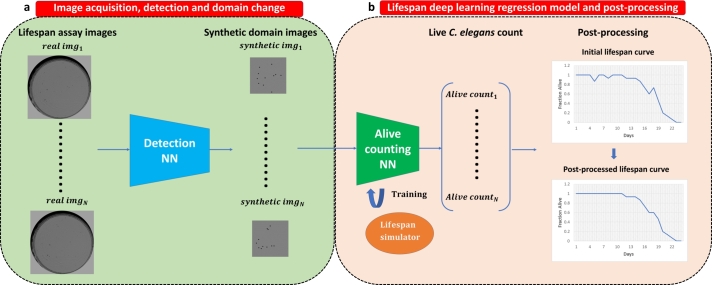
Pipeline of the proposed lifespan automation method: (a) capture, detection and domain change; (b) obtaining the lifespan curve.

This paper proposes a method to automate the lifespan assay with *C. elegans*, that is, to establish the number of live *C. elegans* on each day of the experiment from an image sequence, and subsequently obtain the survival curve. Fig. 1 shows the pipeline of our method, which comprises two stages: First, the image sequence of the assay is captured. This sequence consists of one image from each day of the experiment. Next, a detection network is used to locate the *C. elegans* present in the images. Using these locations, a new type of simplified image is generated, in which circular blobs appear on a constant background at the positions where the *C. elegans* are located. This sequence in synthetic domain is the input of a regression neural network, which returns to the output a vector with the number of live *C. elegans* in each day. As shown in Fig. 1, the synthetic images are rescaled to a lower resolution to train the counting network without causing memory problems. Finally, post-processing is applied to the curve obtained.

### 3.1 Image acquisition method

**Fig. 2:**
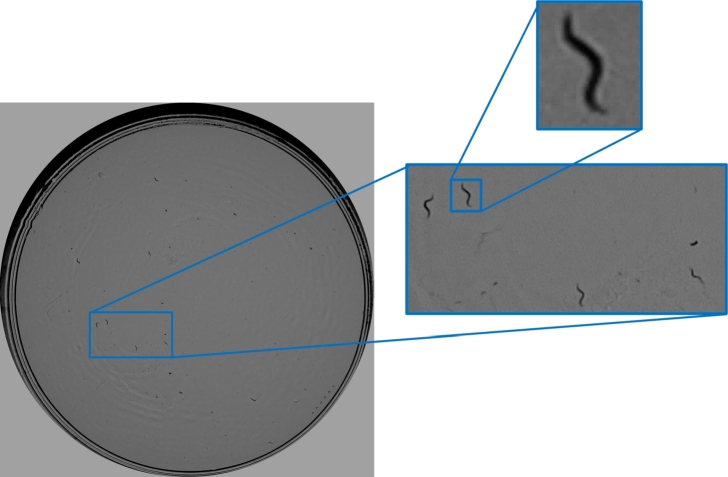
Example of a complete picture of the Petri plate captured with SiViS.

Our lifespan method needs to capture images of the whole Petri dish, for which members of our laboratory have developed two capture systems: (1) a Cartesian multi-view robot [37], which can also analyze images at the micro level and (2) SiViS [13], an open software and hardware system, which has been used in this work. This system uses a backlight configuration, in which a lighting system (a 7” Raspberry Pi display 800 × 480 at a resolution at 60 fps, 24-bit RGB color) is placed at the bottom, while at the top there is a RGB Raspberry Pi camera v1.3 (OmniVision OV5647, which has a resolution of 2592 × 1944 pixels, a pixel size of 1.4 × 1.4 μm, a view field of 53.50° × 41.41°, optical size of 1/4”, and focal ratio of 2.9). In this configuration, the Petri dish is placed in between the two. This system incorporates an intelligent illumination control [38] (performed by a Raspberry Pi 3) that maintains the background and the nematodes in less variable gray ranges, which facilitates segmentation. The distance between the camera and the Petri plate is sufficient to enable a complete picture of the Petri plate, and the camera lens is focused at this distance (about 77 mm). With this image capture and resolution setting (1944 × 1944 pixels), the worm size projects approximately 55 × 3 pixels. An example of the image captured by the SiViS system is shown in Fig. 2.

### 3.2 Domain change method based on detection

Once the image sequences of the lifespan assay have been captured, the next step of the method is the domain change from the real images to the simplified image domain, on which the counting neural network has been trained. As discussed in the related work section, when transforming real images to synthetic ones, the use of generative adversarial networks [39] is common. Analyzing our problem, we concluded that the optimal solution for our case was the use of a detection network for the following reasons:

1. GANs are complicated to train (convergence problems, modal collapse, difficulty in choosing metrics, need for complex cost functions), while training a pre-trained detection network is simpler.
2. Supervised GANs with the paired data method need a lot of data for training, while the detection network can take advantage of pre-training.
3. As discussed in [40], unsupervised GANs tend to work well in style transfer if it involves changes in color and texture. However, if the task requires geometry changes and detection of objects to be removed (in our case the edge of the plate, dirt, and other opaque objects that can be mistaken for the nematode), making it work is even more complex.
4. GANs can generate images with artifacts, while using the detection network to make the domain change will only produce false positive or false negative errors.

**Fig. 3:**
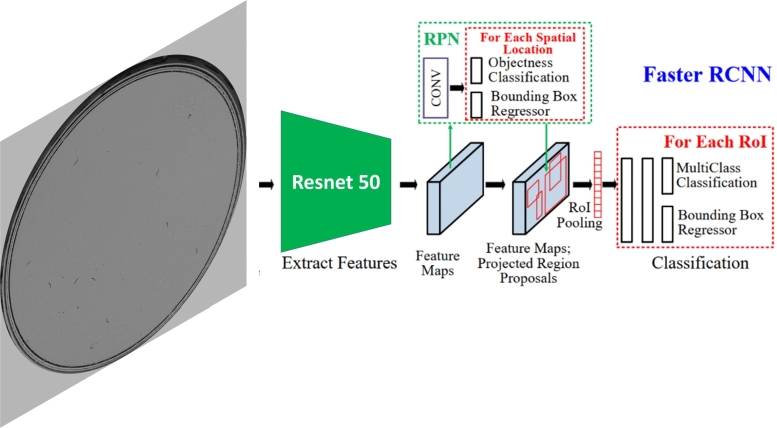
Faster R-CNN detection network architecture employed [44].

In recent years, convolutional neural networks (CNNs) have facilitated great advances in the detection of small objects [41]. Noteworthy are the one-stage (YOLO [15], SSD [42]) and two-stage methods (Faster R-CNN [16], RFCN [43]). The two-stage methods use a Region Proposal Network (RPN), which generates the candidate bounding boxes to be objects of interest. The network then determines the class to which each candidate belongs and performs a regression to refine the bounding box coordinates. Methods such as YOLO perform detection and classification in a single stage. One-stage methods are faster but two-stage methods are more accurate. Therefore, as our application does not require speed, we chose Faster R-CNN. In addition, accuracy and robustness are needed as detection must be performed at different *C. elegans* life stages and on backgrounds with possible dirt on the Petri dish. Specifically, the Faster R-CNN model with a ResNet-50-FPN backbone pre-trained on COCO dataset has been used. The network architecture is shown in Fig. 3.

**Fig. 4:**
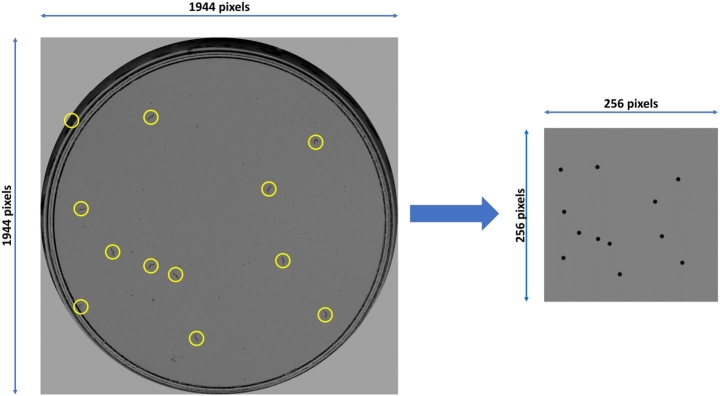
Domain change from real (left) to simplified synthetic image (right).

The model was implemented and trained on a computer with an Intel® Core™ i9-9900KF processor and an NVIDIA GeForce RTX 2080 Ti GPU. The network was trained for 20 epochs with an initial learning rate of 0.005 modified every 3 epochs by a factor of 0.1. The optimizer used was SGD with momentum 0.9 and weight decay 0.0005. Vertical flip was used as a data augmentation technique. As shown in Fig. 4, the real image is processed by the detection neural network obtaining the coordinates of the bounding box for each nematode. Using these coordinates, the synthetic domain image is created, rescaled to a resolution of 256 x 256 pixels. This size is chosen because the aim is to work with the lowest possible resolution in order to train the counting network without incurring memory problems. In our repository (https://github.com/AntonioGarciaGarvi/Celegans-Lifespan-Automation-Using-Deep-Learning), readers can find a demo with some examples of how our model detects *C. elegans* and the synthetic images generated.

### 3.3 Counting method

**Fig. 5:**
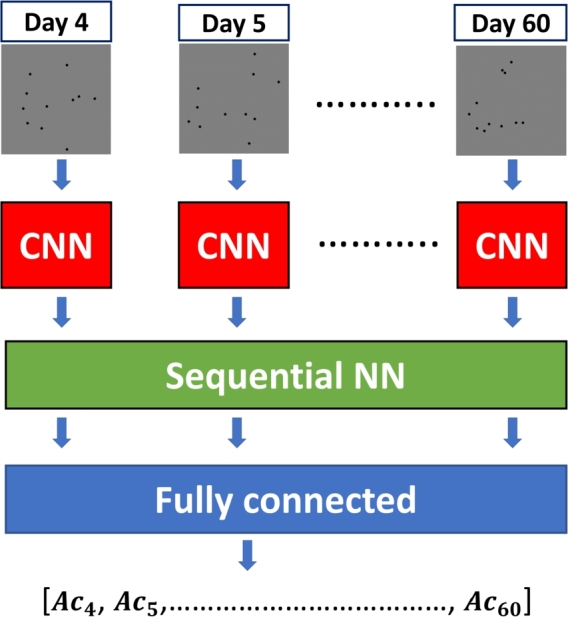
Diagram of the architecture used. The CNN performs the feature extraction, the sequence neural network extracts the temporal information, and the fully connected layers perform the live *C. elegans* counting.

To solve the problem of counting live *C. elegans*, it was approached as a regression in which the input of the network is a sequence of images (1 for each day of the experiment) and the output is a vector with the number of live *C. elegans* in each image. Our method uses a sequence length of 57 images, since the longest lasting assay we perform is 60 days, and counting starts from day 4, which is approximately when *C. elegans* reaches adulthood. The determination of the number of live nematodes for each day is based on the changes in position between days; therefore, it is necessary to use temporal information. Taking this into account, a sequence to sequence architecture was proposed, consisting of three convolutional layers acting as feature extractors, sequential layers extracting the temporal information and finally three linear layers obtaining the count vector. A schematic image of the proposed counting method is shown in Fig. 5. For the sequential processing part, three alternatives were compared: LSTM, GRU and Transformer. This comparison is shown in the results section. Table 2 shows the hyperparameters of the seq2seq models compared. The models were implemented and trained using the Pytorch deep learning framework on a computer with an Intel® Core™ i7-7700K processor and NVidia GeForce GTX 1070 Ti graphics card. The models were trained for 600 epochs with a learning rate of 0.001 and a batch size of 16 samples. The cost function (Eq. (1)) was the mean squared error (MSE) and the optimizer used was SGD.

(1) $$\text{MSE}=\frac{1}{N}\sum_{i=1}^{N}{\left(\overset{ˆ}{y_{i}}-y_{i}\right)}^{2}$$

where N is the total number of frames in the batch, $\overset{ˆ}{y_{i}}$ and $y_{i}$ are the model prediction for the number of live *C. elegans* in frame i and the true number of live *C. elegans* in frame i, respectively (Table 1).

**Table 1:** Summary of the alive counting neural network architecture used.

Layer name	Output size	Layer details
conv1	[Batch size x seq length, 4, 252x252]	5x5, 4, stride 1
cnn bn1	[Batch size x seq length, 4, 252x252]	*eps* = 1*e* − 05, momentum=0.1
leaky relu1	[Batch size x seq length, 4, 252x252]	-
max pooling1	[Batch size x seq length, 4, 84x84]	3x3, stride 3
conv2	[Batch size x seq length, 8, 80x80]	5x5, 8, stride 1
cnn bn2	[Batch size x seq length, 8, 80x80]	*eps* = 1*e* − 05, momentum=0.1
leaky relu2	[Batch size x seq length, 8, 80x80]	-
max pooling2	[Batch size x seq length, 8, 20x20]	4x4, stride 4
conv3	[Batch size x seq length, 16,16x16]	5x5, 16, stride 1
cnn bn3	[Batch size x seq length, 16, 16x16]	*eps* = 1*e* − 05, momentum=0.1
leaky relu3	[Batch size x seq length, 16, 16x16]	-
max pooling3	[Batch size x seq length, 16, 8x8]	2x2, stride 2
seq2seq	[Batch size, seq length, 1024]	See Table 2
linear1	[Batch size, 2000]	In features = seq length x 1024 Out features = 2000
bn fc 1	[Batch size, 2000]	*eps* = 1*e* − 05, momentum=0.1
leaky relu fc1	[Batch size, 2000]	
linear2	[Batch size, 2000]	In features = 2000 Out features = 2000
bn fc2	[Batch size, 2000]	*eps* = 1*e* − 05, momentum=0.1
leaky relu fc2	[Batch size, 2000]	
linear3	[Batch size, 750]	In features = 2000 Out features = 750
bn fc 3	[Batch size, 750]	*eps* = 1*e* − 05, momentum=0.1
leaky relu fc3	[Batch size, 750]	
linear4	[Batch size, seq length]	In features = 750 Out features = seq length

**Table 2:** Hyperparameters of the seq2seq models used.

LSTM & GRU		TRANSFORMER				
Hidden size	Num layers	dim	depth	heads	mlp dim	dim head
1024	2	1024	2	8	2048	64

#### 3.3.1 Simulator

As mentioned in the introduction, training neural networks may require a large amount of data depending on the task to be solved. For this reason, the use of synthetic data has been proposed in this paper. To avoid the domain gap problem, we decided to transform the real image to a simplified domain and then train the lifespan network with these synthetic images. The simulator uses a mathematical model to generate the lifespan curves, and in this case we have used the Weibull model (Eq. (2)) [45]. Weibull model can calculate the percentage of *C. elegans* alive on the day of experiment t, using two parameters (a, b) as input, which are related to the slope of the mortality curve and the mean lifespan, respectively. Random transformations are applied to these theoretical curves to avoid overfitting the neural network to them. In addition, during the simulation, cases like those occurring in the real images are recreated: (a) occlusions causing blobs to disappear on one day and reappear on the following days; (b) on days without capture (weekends and holidays) a blank image is used; (c) small rotations and translations that may occur when placing the plates in the capture system. Using the generated curve, images are generated for each day of the experiment, as shown in Algorithm 1.

(2) $$survival(t)=e^{\frac{-t}{b}a}$$

**Algorithm 1:**
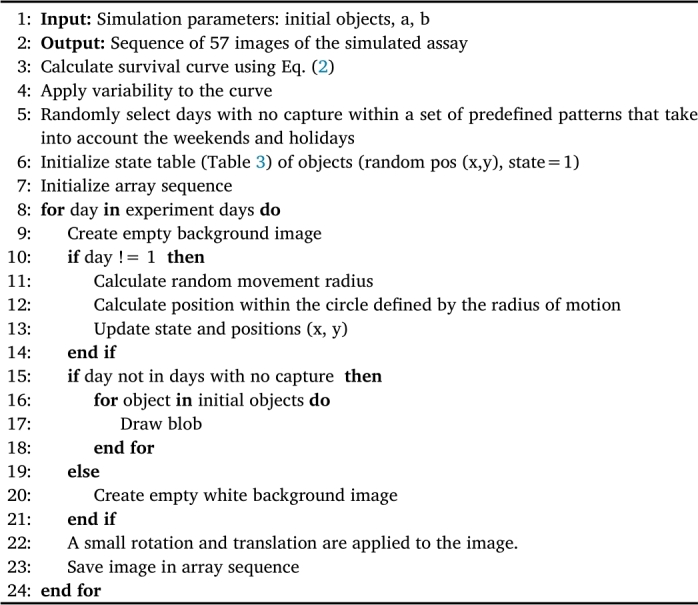
Simplified domain image simulator.

The simulation is done online, i.e., the dataset is generated during training, thus avoiding the need to store the images on disk. The image size is small (256 x 256 pixels) and the operations are simple; therefore it can be simulated quickly. The input parameters to the simulator are the mean life, the slope and the number of objects to simulate. Thus, the sequence generated by the simulator for each combination of these parameters is an input to the network. The number of objects varies between 10 and 15, since this is the number of *C. elegans* that are usually present in the experiments in our dataset. The parameter affecting the mean life oscillated between 5 and 57, and the slope parameter between 4 and 20. This combination gives rise to 5406 theoretical curves, which are also randomly modified, so they change in each training iteration. This, together with the fact that the positions of the blobs is also randomly generated each time, increases the variability, making the dataset as large as required.

**Table 3:** Simulation state table. ID is the identifier of each of the N blobs. State is 0 if the blob is dead or 1 if it is alive. Last position is the coordinates (x,y) where the blob was in the previous day's image.

ID	State: 0 (dead) / 1 (alive)	Last position (x, y)
1	st1	X1, Y1
.	.	.
.	.	.
N	stN	XN, YN

As our lifespan regression model has a fixed sequence length (57), when experiments are of shorter duration, we use padding which consists of replicating the last image until the end of the sequence. Therefore, as there are no worm displacements, the neural network must learn that all worms are dead. Additionally, we use a different type of padding on days when no images are captured. In such cases, we insert blank images as padding. This ensures that the temporal continuity of the experiment is maintained in the input data while allowing the neural network to recognize periods when no images were available for analysis. These blank image paddings do not affect the real-time intervals either, as they represent the absence of data rather than artificial worm activity.

#### 3.3.2 Postprocessing

The last step involves correction of the obtained curves, since the lifespan curves must decrease monotonically. The post-processing method proposed in [46] is applied. This method divides the curve into two cycles using the mean life of the strain (usually 14 days for strain N2 and 42 for *daf-2*). In the first cycle, which corresponds to the days with the highest probability of occlusions or aggregations, if the number of nematodes is higher on one day than on the previous one, the latter is corrected upwards. The second cycle corresponds to days with less movement and greater accumulation of dirt, which leads to false detections, so if more nematodes are detected in the current day's count than on the previous day, it is corrected downwards. In our repository (https://github.com/AntonioGarciaGarvi/Celegans-Lifespan-Automation-Using-Deep-Learning), readers can find an example of how our model predicts lifespan curve given the simplified domain images as input.

## 4 Experiments and results

### 4.1 Lifespan assay protocol

*Caenorhabditis elegans* strains used were: N2, Bristol (wild-type), and CB1370, *daf-2* (*e1370*), maintained under standard conditions at a temperature of 20 °C. All nematodes were age-synchronized and pipetted onto Nematode Growth Medium (NGM) in 55 mm Petri plates. Several problematic issues were considered when performing the assay: (1) nematode reproduction was prevented by adding FUdR (0.2 mM) to the plates; (2) fungal contamination was reduced by adding Fungizone(1 μg/mL) [47]; (3) nematode occlusion, caused by worms climbing up the plate walls, was reduced by placing the food (OP50 strain of *Escherichia coli*), in the middle of the Petri dish. The experiment was carried out by a laboratory technician from the first day until the last nematode died. Each day, the plates were removed from the incubator, placed in the monitoring system and checked to ensure there was no condensation on the lid. Then, a sequence of 30 images at 1 fps was captured and once this was completed, the plates were returned to the incubator.

### 4.2 Datasets

Following the protocol described above, images were captured from 106 plates, each containing between 10 and 15 nematodes. Part of the images were labeled for the detection problem and others for the live and dead counts. The labeling of the lifespan dataset was performed by visualizing the sequence of 30 captured images and identifying the *C. elegans* in it, for each day. To distinguish them from other possible objects in the image, analyses were made of the characteristics related to color, length and type of movement. Once the *C. elegans* had been identified, they were marked (at their centroid approximately) as alive or dead depending on their movement. If they moved during the sequence, they were considered alive. If they did not move, their position and posture were compared with the images of the previous and following day. If they remained the same as on those two days, they were considered dead, otherwise they were marked as alive. The images of the detection dataset were labeled analogously, marking the centroid. The images were then processed to obtain the bounding-box labels. Using the marked centroid, a window was generated around it. Working with this sub-image, we segmented it by applying a threshold of 33, we obtained the contour and the minimum rectangle containing it, which gave the coordinates of the bounding box. Finally, the generated bounding box was checked for correctness and in case of a segmentation error, it was manually corrected. Our proposed method's annotations were generated through consensus reached by a small group of human annotators, resulting in no variability between independent annotators. As can be seen, this procedure is very laborious, hence, the cost of generating a labeled dataset is high.

### 4.3 Results of detection network

The set of images made available the detection network (a total of 1900) was divided into 80% for training and 20% for validation. In this section we show the results obtained in the validation dataset using common object detection metrics [48]. To determine whether a prediction is correct, the Intersection over Union (IoU), which evaluates the overlap between two bounding boxes, was used. It is calculated by dividing the area of overlap between the actual and the predicted bounding box by the area of union of the two (Eq. (3)):

(3) $$\text{IoU}=\frac{\text{area of overlap}}{\text{area of union}}$$

An IoU threshold of 0.5 was set so that:

• If the IoU is greater than or equal to the threshold, it is a correct detection, True positive (TP).
• If the IoU is less than the threshold, it is an incorrect detection, False positive (FP).
• When an object is not found by the network, it is counted as False negative (FN).

Once the predictions were evaluated, the following metrics were used:

• Precision (Eq. (4)). It is the percentage of correct detections out of the total number of detections made by the network.
• Recall (Eq. (5)). It is the percentage of objects found by the network.
• F1 score (Eq. (6)). It is a harmonic average of precision and recall.

(4) $$\text{Precision}=\frac{\text{TP}}{\text{TP + FP}}=\frac{\text{TP}}{\text{all detections}}$$

(5) $$\text{Recall}=\frac{\text{TP}}{\text{TP + FN}}=\frac{\text{TP}}{\text{all ground truths}}$$

(6) $$\text{F1 score}=\frac{\text{2 ⋅Precision ⋅Recall}}{\text{Precision + Recall}}$$

The detection network returns the predictions together with a prediction confidence score. Using this score we performed a filter, keeping only those predictions that were above a threshold. Table 4 shows the results obtained by the detection network for different threshold values in the validation dataset. Finally, we chose to use a threshold of 0.85 for generating the synthetic images, since it presented a better balance between precision and recall.

**Table 4:** Results obtained by the detection network in the validation dataset for different score thresholds.

Scores threshold	Targets	Detections	TP	FP	FN	Precision	Recall	F1 score
0.95	4037	3436	3195	241	842	0.929	0.791	0.855
0.9	4037	3764	3363	401	674	0.893	0.833	0.862
0.85	4037	3971	3447	524	590	0.868	0.854	0.861
0.8	4037	4127	3494	633	543	0.847	0.865	0.856
0.7	4037	4398	3567	831	470	0.811	0.884	0.846

### 4.4 Lifespan results

In order to analyze method robustness, the lifespan method was validated with two assays using different strains: one with short-lived *C. elegans* (N2) and the other with a long-lived strain (*daf-2*). The aim of the experiment was to compare the curves obtained by the proposed method with the manually labeled curves following the procedure described in the dataset section and to calculate the errors. Errors were calculated as follows in the same way as in [31]: (1) the image sequences for each of the assay plates were processed using the proposed method, thus obtaining the count for each day of the experiment; (2) for each day, the number of live *C. elegans* was added up and the survival percentage was obtained by dividing by the number of initial nematodes (Eq. (7)); (3) the error for each day (Eq. (8)) was obtained as the difference in absolute value of the percentage survival of the manual and the automatic curve; (4) finally the mean and standard deviation of the errors were calculated (Eq. (9)).

(7) $$\text{\% live}\;\text{C. elegans}=\frac{\text{live}\;\text{C. elegans}\;\text{current day ⋅100}}{\text{initial live}\;\text{C. elegans}\;}$$

(8) e(d)=|%livemanual(d)−%liveautomatic(d)|

(9) $$\text{MAE}=\frac{\sum_{d=1}^{days}e(d)}{days}$$

For the experiment with strain N2, an assay was performed with n=103 nematodes, distributed among 10 test plates, with approximately 11 *C. elegans* per plate.

The experiment with strain *daf-2* was performed using 4 test plates, each containing 13 nematodes, resulting in a total of n=52 nematodes.

#### 4.4.1 Architecture comparison

In this experiment a comparison was made between the results of different SOTA architectures for sequence processing. Specifically, the LSTM, the gated recurrent unit (GRU) and the Transformer were evaluated. Since the initialization of the weights influences the results, three training sessions were performed with each alternative, calculating the error and standard deviation using the validation method explained in this section (Eq. (9)). Subsequently, the average of the mean errors and the average standard deviation of the trained models were calculated. Based on these two metrics, the models were compared, determining which model obtained the best results, and whether the differences were statistically significant. The results of each of the test replicates have been included in Appendix A.

Table 5 shows the average results of the trials. In Appendix B, we report a MANOVA analysis performed to test whether architecture significantly influences the error metrics. The results indicate that architecture does not have a statistically significant effect on the error metrics.

**Table 5:** Average results of the 3 trials performed with each model. The mean and standard deviation of the MAE (%) are presented.

CNN-LSTM		CNN-GRU		CNN-Transformer	
Mean	std	Mean	std	Mean	std
4.98	4.35	5.06	4.03	4.29	3.86

#### 4.4.2 Lifespan statistical analysis results

**Fig. 6:**
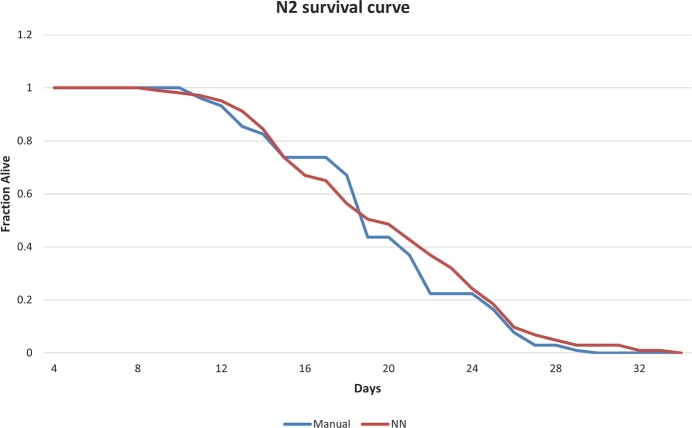
Manual-automated comparison for N2 strain. The horizontal axis shows the days of the experiment, and the vertical axis shows the proportion of live *C. elegans*.

This section shows the results of the statistical study of the model (CNN-Transformer) that obtained the best results in the previous comparison. Fig. 6 shows the results obtained for N2 assay. The mean error obtained was 3.32 ± 3.67%.

**Fig. 7:**
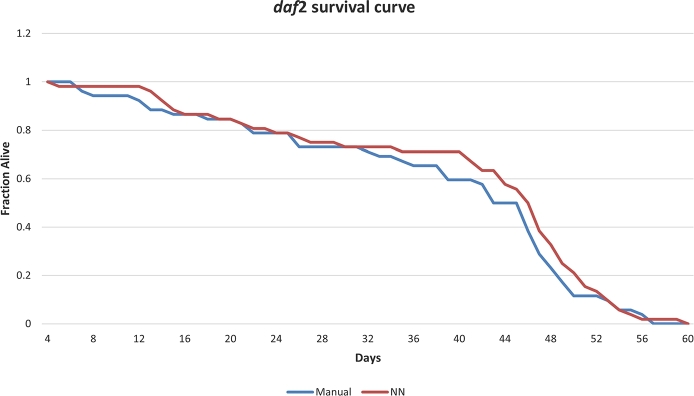
Manual-automated comparison for *daf-2* strain. The horizontal axis shows the days of the experiment, and the vertical axis shows the proportion of live *C. elegans*.

The results obtained for *daf-2* assay are shown in Fig. 7. The mean error obtained was 3.74 ± 3.55%.

Once the curves were obtained, statistical significance was evaluated using the open-source tool OASIS [49]. The results obtained (Appendix C) show that the differences between the curves obtained with the proposed method and the manual curves were not statistically significant for either short-lived N2 (p-value log rank test 0.45) or long-lived *daf-2* (p-value log rank test 0.83) strains. In addition to the log-rank test, three statistical tests, the survival time F-test, partial slopes rank-sum test and normalized chow test, have been added to identify the differences in lifespan variations.

## 5 Discussion and conclusions

**Fig. 8:**
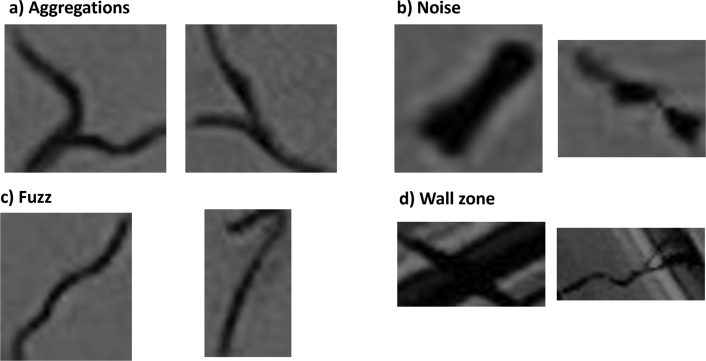
Examples of error cases of the detection network. a) Errors due to *C. elegans* aggregation. b) Cases of dark blobs (noise) appearing on the plate. c) Examples of blobs with nematode-like characteristics. d) Errors in the edge zone of the plate.

This paper has presented a new method for automating the *C. elegans* lifespan assay using deep learning. To solve the problem, a training strategy has been proposed for cases where few data are available. Following this strategy, we have divided the task of counting of live *C. elegans* into two stages. The first stage involves the detection and transformation from the real to the simplified domain, whereas the second one involves counting by regression. Despite having few training data, good results are obtained with the Faster R-CNN detection network (f1-score 0.86), as most of the errors are due to cases where errors in capture or plate soiling occurred. Examples of errors made by the detection network are shown in Fig. 8. We find cases of nematode aggregation, where instead of detecting two individuals, the model detects them as if they were one. One possible cause of this error is that there are very few cases of this situation in the dataset, because it hardly occurs in the assay when working with few nematodes per plate. This problem means the method is not robust for assays with high-aggregating strains, such as the Hawaii-type strains. Another typical error occurs with dark objects that have similar characteristics to *C. elegans*, such as fuzz. Some errors also appear at the edge of the plate, as this is a poorly illuminated area and thus hinders establishing whether the object detected is a nematode or not. These cases usually require human analysis of a temporal sequence, since, as mentioned in the previous section, labeling is done by analyzing a sequence of 30 images. Therefore, considering that the detection network solves the problem using a single image, this is an expected error. Moreover, as in the case of aggregation, there are not many cases of this type in the dataset either. To demonstrate that our results are independent from the specific splitting of the data set, we performed a 5-fold cross-validation. The results of this experiment confirm the consistent performance of the model across different subsets (see detailed results in Appendix D).

**Fig. 9:**
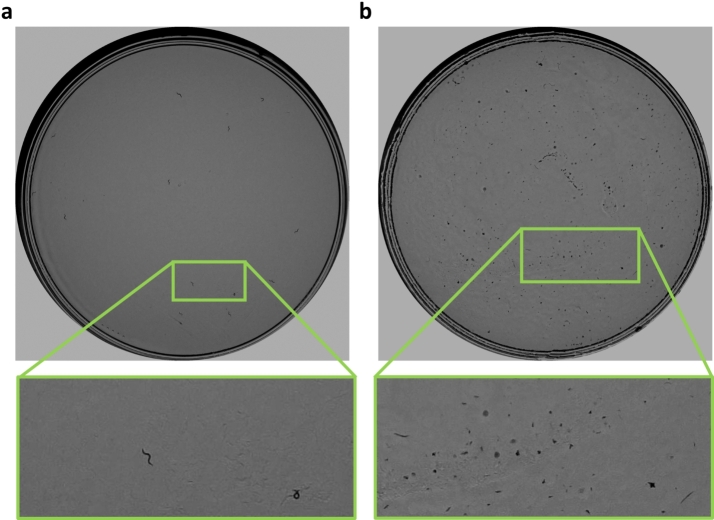
Temporal changes in the dirt on the plates: a) example of a plate from the first days of the assay and b) example of an image from the last days of the assay.

The lifespan method has been validated with N2 and *daf-2* strains, obtaining curves with statistically non-significant differences with respect to those labeled manually. However, the method presents some errors. As expected, the experiment with the *daf-2* strain presents a higher error due to the longer experimental duration, which causes dirt accumulation on the plates. Fig. 9 shows a comparison between a plate in the early days and one in the later days. This gives rise to a higher number of errors in detection and, therefore, also in counting. Another aspect to consider in these long experiments is that there are more cases of *C. elegans* remaining stationary in the last days, making only small head and tail movements. Such movements are more difficult to detect with our method, as it uses low resolution. Errors have been corrected slightly by applying the post-processing technique. One of the problems encountered by the model concerns prediction on days without image capture (holidays and weekends), on which the manual curve maintains the count of the previous day. To avoid these discrepancies, the predicted curve could be modified, since the days without image captures are known or, alternatively, the real curve could be modified by interpolating instead of keeping the *C. elegans* count from the previous day.

When comparing our lifespan automation method with existing methods [8], [10], [11], [12], [46], we found that the algorithms employed by their software are tuned for images captured with their specific system (resolution, illumination conditions, lifespan protocol), therefore, a quantitative comparison is not possible. Likewise, our model is trained to work with images captured with our acquisition system, and cannot be directly applied to images captured by the other devices. To do so, the model would need to be adapted and retrained. Therefore, of all these traditional methods, we were only able to make a direct comparison with our previous method [46], which is designed for our capture conditions. All traditional methods perform similar processing steps (alignment and differentiation between images) to determine the death of *C. elegans*. Therefore, this is indicative of how our method would work compared to the others. The results (see appendix E) show how the proposed regression model obtains error rates similar to those of the traditional automatic method in the lifespan curves and how the detection models allow better filtering of noise when detecting worms.

The approximate cost of labeling a 30-day assay is 13 working days per condition (approx. 10 plates). To this cost must be added the work to be done for the preparation of the assay plates and the time spent by the laboratory technician to capture the images, which vary between 30 and 60 days depending on the strain (N2 and *daf-2*). Taking into account this cost, the use of synthetic data represents a great saving of time and costs.

Considering the initial limitations in terms of the lack of data and computational resources, the results (Sect. 4.4.2) are quite good. Future work could improve the results by improving the detection network, exploring different architectures and hyperparameters. In Appendix D we have added a trial with a Faster R-CNN model employing a Resnet50-FPN backbone version 2 to show how using models with more parameters can improve detection results. The results could also be improved by trying to simulate possible errors in the detection network of the lifespan simulator. In this work we have focused on recreating the cases that occur in real images (occlusion, rotation and translation and days without capture). This strategy has enabled us to solve the problem of counting live *C. elegans* and provides us with the nematode's location, making this network useful to solve tracking problems too. This strategy could also be extrapolated to resolving other animal-behavior monitoring problems (trajectory analysis, anomaly detection), in which obtaining large labeled datasets is laborious and time consuming.

## CRediT authorship contribution statement

**Antonio García-Garví:** Conceptualization, Methodology, Software, Validation, Data curation, formal analysis, investigation, Writing – original draft, writing—review and editing. **Pablo E. Layana-Castro:** Software, Visualization, Writing—review and editing. **Joan Carles Puchalt:** Software, Visualization, Writing—review and editing. **Antonio-José Sánchez-Salmerón:** Conceptualization, Methodology, Writing – original draft, writing—review and editing, Investigation, Resources, Project administration, Funding acquisition.

## Declaration of Competing Interest

None declared.

## Figures and Tables

**Table A.1:** Summary of results trial 1. The MAE (%) and standard deviation are presented.

Strain	CNN-LSTM		CNN-GRU		CNN-Transformer	
	MAE	std	MAE	std	MAE	std
N2	3.85	2.84	4.82	4.71	3.76	4.38
*daf-2*	5.19	5.19	7.09	5.02	5.94	3.97
Average	4.52	4.02	5.95	4.87	4.85	4.17

**Table A.2:** Summary of results trial 2. The MAE (%) and standard deviation are presented.

Strain	CNN-LSTM		CNN-GRU		CNN-Transformer	
	MAE	std	MAE	std	MAE	std
N2	3.13	4.15	3.45	2.71	3.70	4.07
*daf-2*	7.59	5.45	6.98	4.66	5.26	3.51
Average	5.36	4.80	5.22	3.69	4.48	3.79

**Table A.3:** Summary of results trial 3. The MAE (%) and standard deviation are presented.

Strain	CNN-LSTM		CNN-GRU		CNN-Transformer	
	MAE	std	MAE	std	MAE	std
N2	4.54	4.04	4.35	4.07	3.32	3.67
*daf-2*	5.60	4.44	3.68	3.02	3.74	3.55
Average	5.07	4.24	4.02	3.55	3.53	3.61

**Table A.4:** Average results of the 3 trials performed with each model. The error per plate (average mean and average standard deviation of the MAE (%)) is presented.

CNN-LSTM		CNN-GRU		CNN-Transformer	
Avg Mean	Avg std	Avg Mean	Avg std	Avg Mean	Avg std
9.20	3.18	9.07	2.66	8.92	3.50

**Fig. B.1:**
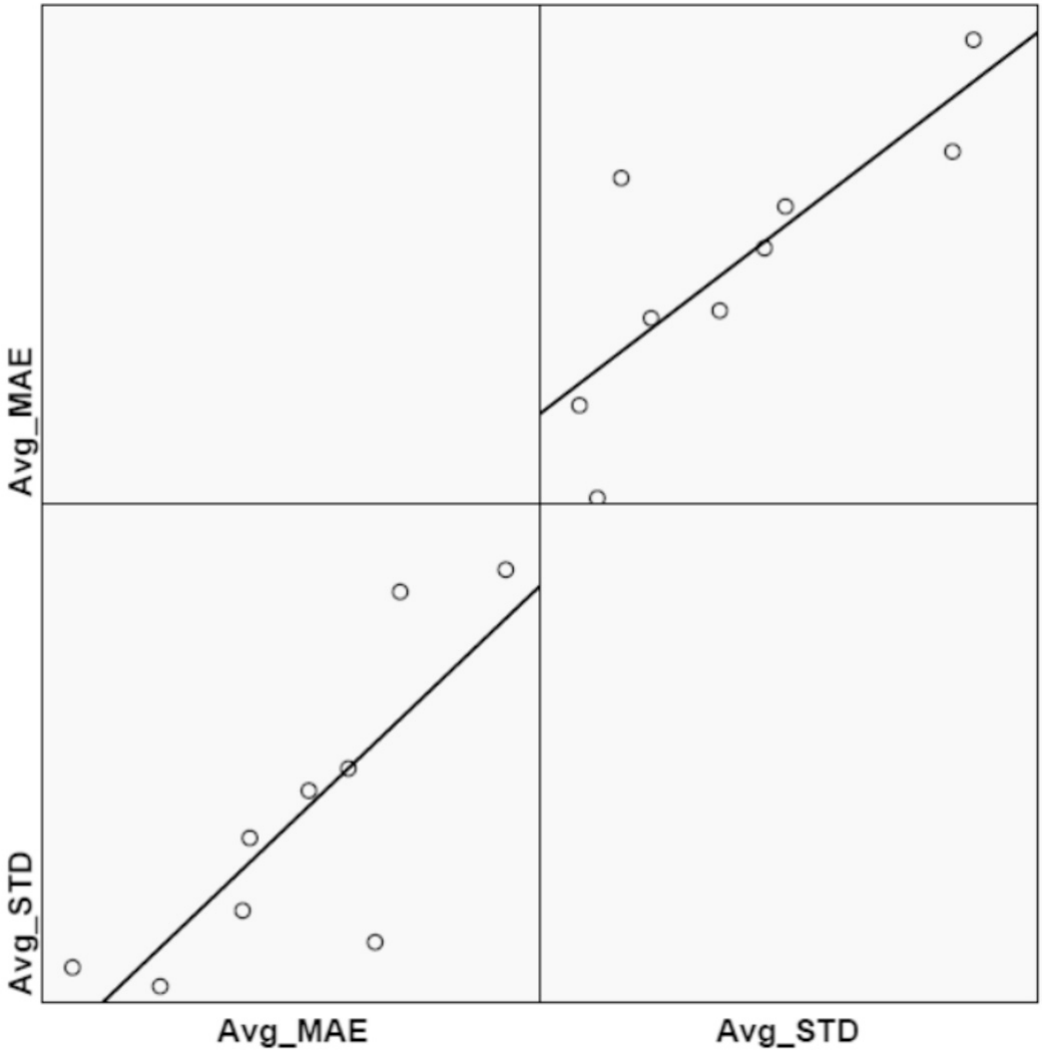
Screenshot of the scatter plot performed with SPSS for the analysis of linearity.

**Fig. B.2:**
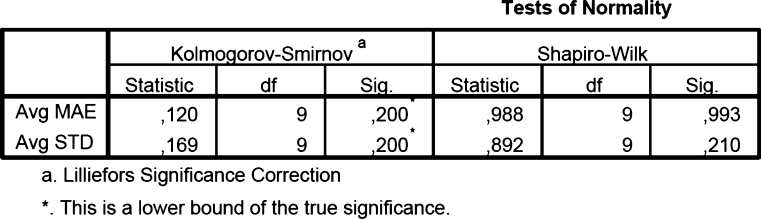
Screenshot of the normality test performed with SPSS.

**Fig. B.3:**
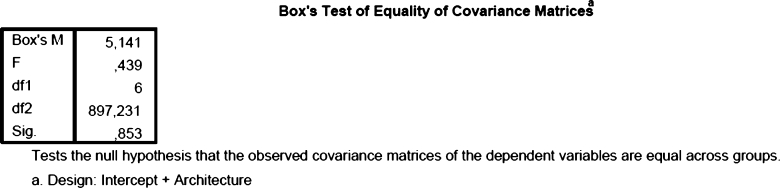
Screenshot of the Box's test for homogeneity of covariance matrices performed with SPSS.

**Fig. B.4:**
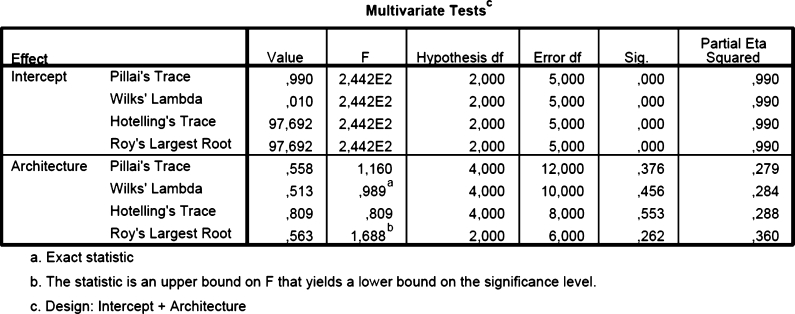
Screenshot of the Multivariate test performed with SPSS.

**Fig. C.1:**
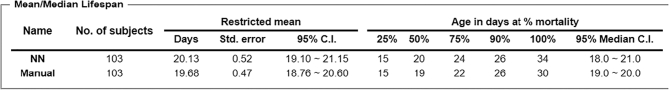
Screenshot of the survival analysis performed with the open source tool OASIS for the N2 strain assay. It shows the Irwin's restricted mean lifespan and the age in days at different % mortality.

**Fig. C.2:**
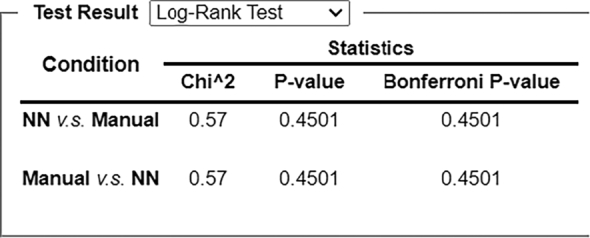
Screenshot of the Log-rank test performed with the open source tool OASIS for the N2 strain assay.

**Fig. C.3:**
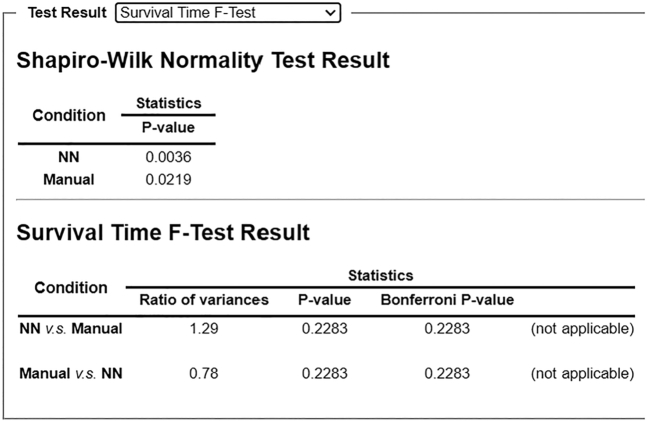
Screenshot of the Survival F-test performed with the open-source tool OASIS for the N2 strain assay. Survival F-test is not applicable because the neural network data do not follow a normal distribution according to the Shapiro-Wilk normality test.

**Fig. C.4:**
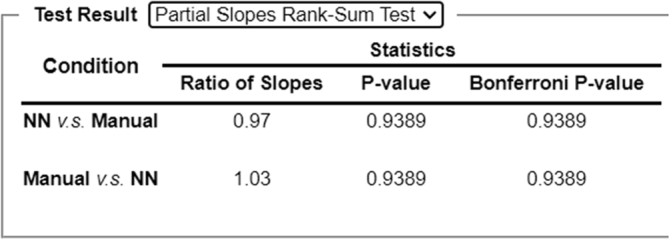
Screenshot of the Partial slopes rank-sum test performed with the open-source tool OASIS for the N2 strain assay. According to the test, as *p-value* > *0.05*, the differences between the slopes are not statistically significant.

**Fig. C.5:**
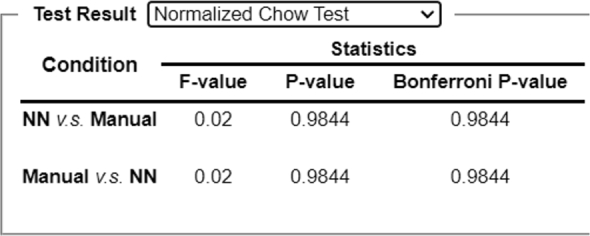
Screenshot of the Normalized Chow test performed with the open-source tool OASIS for the N2 strain assay. According to the test, as *p-value* > *0.05*, the differences between the slopes are not statistically significant.

**Fig. C.6:**
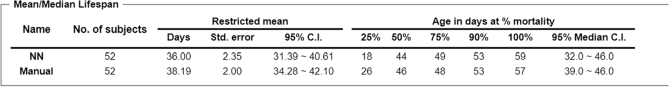
Screenshot of the survival analysis performed with the open-source tool OASIS for the *daf-2* strain assay. It shows the Irwin's restricted mean lifespan and the age in days at different % mortality.

**Fig. C.7:**
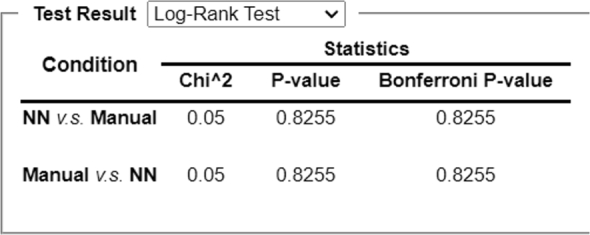
Screenshot of the Log-rank test performed with the open-source tool OASIS for the *daf-2* strain assay.

**Fig. C.8:**
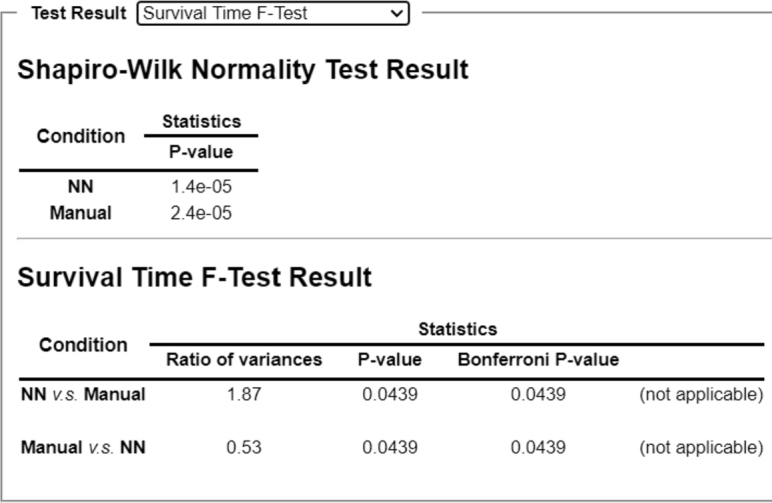
Screenshot of the Survival F-test performed with the open-source tool OASIS for the *daf-2* strain assay. Survival F-test is not applicable because the neural network data do not follow a normal distribution according to the Shapiro-Wilk normality test.

**Fig. C.9:**
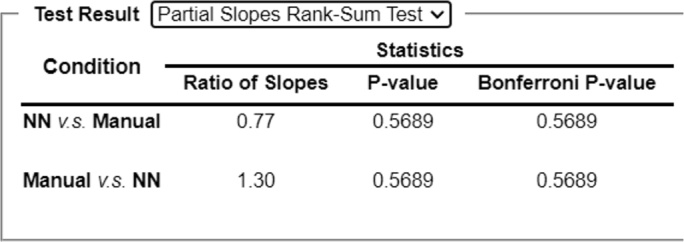
Screenshot of the Partial slopes rank-sum test performed with the open-source tool OASIS for the *daf-2* strain assay. According to the test, as *p-value* > *0.05*, the differences between the slopes are not statistically significant.

**Fig. C.10:**
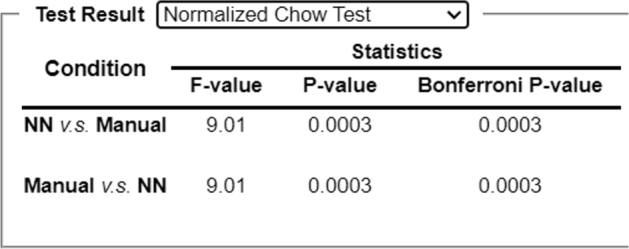
Screenshot of the Normalized Chow test performed with the open-source tool OASIS for the *daf-2* strain assay. According to the test, as *p-value* < *0.05*, the differences between the slopes are statistically significant.

**Fig. C.11:**
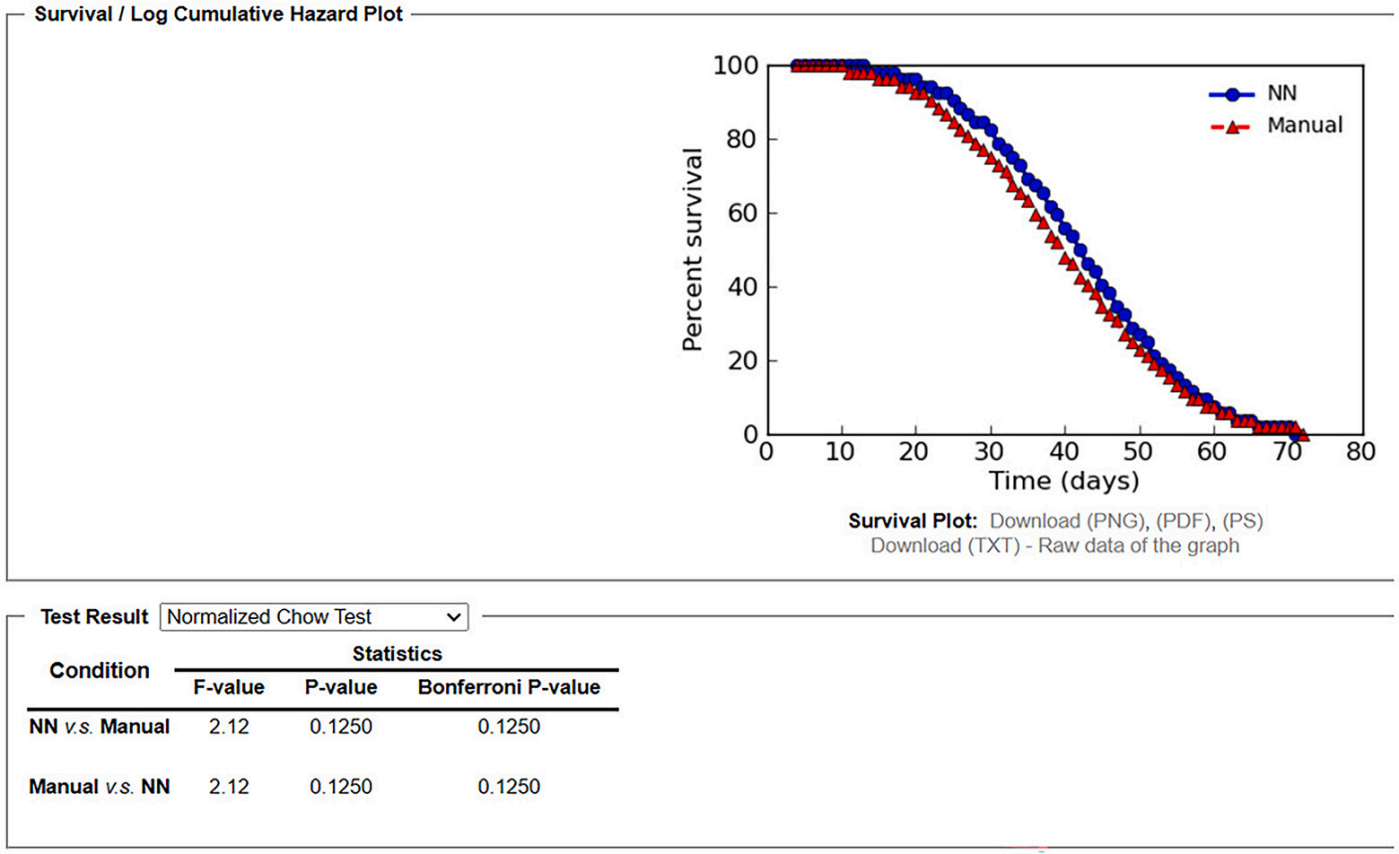
Screenshot of the Normalized Chow test performed with the open-source tool OASIS for the Weibull fit of the *daf-2* strain assay.

**Table D.1:** Results of the 5-fold cross-validation for the Faster R-CNN with a Resnet50-FPN backbone.

	Fold 1	Fold 2	Fold 3	Fold 4	Fold 5	Mean	Std
mAP@[0.5–0.95]	0.5555	0.5535	0.5721	0.5638	0.5688	0.5628	0.0081

**Table D.2:** Results of the 5-fold cross-validation for the faster R-CNN model with a ResNet-50-FPN backbone version 2.

	Fold 1	Fold 2	Fold 3	Fold 4	Fold 5	Mean	Std
mAP@[0.5–0.95]	0.6039	0.6045	0.6226	0.6169	0.6150	0.6126	0.0082

**Table E.1:** Detection results obtained with the Faster R-CNN model.

	Faster R-CNN							
	TP	FP	FN	Detections	Targets	Precision	Recall	F1 score
*daf*-2	1324	126	166	1450	1490	0.913	0.889	0.901
N2	1624	144	86	1768	1710	0.919	0.950	0.934

**Table E.2:** Detection results obtained with the traditional method.

	Traditional method							
	TP	FP	FN	Detections	Targets	Precision	Recall	F1 score
*daf*-2	1203	706	286	1909	1489	0.630	0.807	0.708
N2	1525	728	183	2253	1708	0.677	0.892	0.770

**Table E.3:** Comparison of lifespan metrics between the traditional method and the neural network model.

	Regression NN		Traditional method	
	Mean	Std	Mean	Std
*daf*-2	3.74%	3.55%	6.04%	6.46%
N2	3.32%	3.67%	1.35%	2.06%
Average	3.53%	3.61%	3.70%	4.26%

**Fig. E.1:**
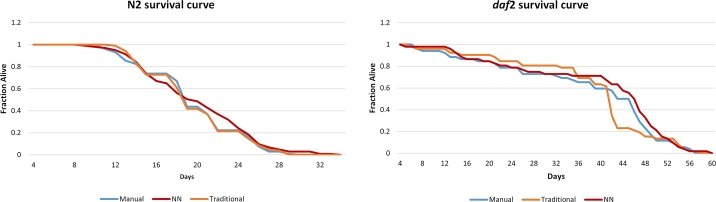
Comparison of manual counting (blue curve), automatic counting traditional method (orange curve), and counting with the proposed method (red curve). The horizontal axis shows the days of the experiment, and the vertical axis shows the proportion of live *C. elegans*.

**Fig. F.1:**
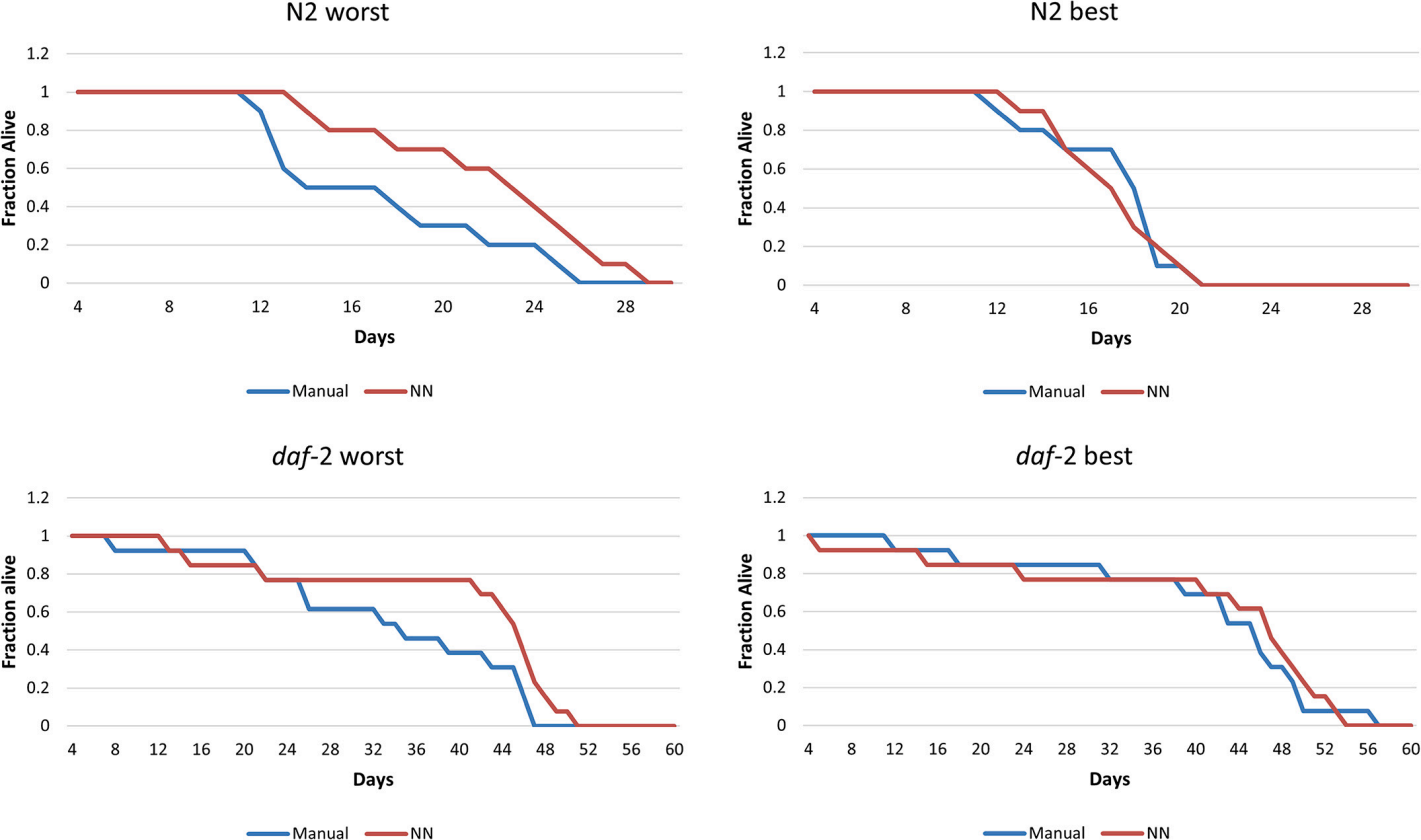
Best and worst curves predicted by our model.

## Data Availability

Our trained models can be downloaded from https://active-vision.ai2.upv.es/wp-content/uploads/2022/03/models.zip. We created a repository on github with a demo of our method: https://github.com/AntonioGarciaGarvi/Celegans-Lifespan-Automation-Using-Deep-Learning (accessed on August 2023). The code, components and the guidelines to build the monitoring system and the assembly description can be found in the repository https://github.com/JCPuchalt/SiViS.

## Appendix A Repeated trials results

This appendix shows the results of each of the test replicates performed in Sect. 4.4.1. The results are shown broken down by each validation test (N2 and *daf-2*) in Table A.1, Table A.2, Table A.3.

The following table shows the average error per petri dish (Table A.4).

## Appendix B MANOVA

The goal of this MANOVA analysis is to determine whether the architecture used (CNN-LSTM, CNN-GRU and CNN-Transformer) significantly affects the accuracy metrics chosen to assess lifespan error (Average MAE and Average STD). Therefore, the independent variable is architecture and the dependent variables are Avg MAE and Avg STD. SPSS 16.0 software was used to perform this analysis. First, before extracting conclusions, it was verified that the assumptions for applying MANOVA were met:

• Independence of observations. Models were trained with different random weight initializations, therefore, observations are independent.
• Linearity. It was checked using a scatter plot (Fig. B.1).
• Normality. We used Shapiro-Wilk test. As shown in the Fig. B.2, the significance values are greater than 0.05. This is an indication that multivariate normality is met.
• Homogeneity of variance-covariance matrices. As can be seen in the Fig. B.3, Box's test for homogeneity of covariance matrices indicates that there is no difference between the covariance matrices (F = 0.439; p-value greater than 0.05).

Once the assumptions were verified, we performed a multivariate test (Fig. B.4). SPSS performs different statistical tests to determine the significance and effect of the independent variable (architecture) on the dependent variables (Avg MAE and Avg STD). Since the p-value is greater than 0.05 for all tests, we can conclude that architecture has no statistical significance on MAE and STD.

## Appendix C Statistical analysis

This appendix shows the screenshots of the statistical study performed with the open source tool OASIS [49] (Figs. Fig. C.1, Fig. C.2, Fig. C.3, Fig. C.4, Fig. C.5, Fig. C.6, Fig. C.7, Fig. C.8, Fig. C.9, Fig. C.10, Fig. C.11).

A possible cause for this could be the steepness of the curves, which is an artefact generated by weekends and holidays. On applying filters, such as fitting the data to theoretical models (Weibull), the results would be as follows.

## Appendix D Additional detection experiments

We conducted a 5-fold cross-validation experiment to assess the performance of our worm detection neural network across different subsets of the dataset. The results, reported as the average ± standard deviation of COCO mean Average Precision (mAP) for thresholds from 0.5 to 0.95 with a step size of 0.05 using pycocotools, are presented in Table D.1.

In addition, we compared its performance with an alternative neural network model. Specifically, it was compared to a more recent version of the initial model, the faster R-CNN with a Resnet50-FPN backbone version 2. This version has a larger number of parameters (43.7 M versus 41.8 M). It also includes improvements in the pretraining methodology. The results are presented below using the same metrics and the same training and validation splits (Table D.2).

## Appendix E Comparison with a traditional method

The proposed method was compared with our traditional method [46] has been carried out. To perform this comparison we have analysed the two parts of the method: detection and regression. First, we compared the accuracy of *C. elegans* detection using the metrics described in section 4. It should be noted that the traditional algorithm does not detect worms, but detects moving objects instead. Therefore, its results do not reflect the detections in one image but the integration of the detections of possible worms in a sequence of 30 images. Filters are then applied to these trajectories (integration of the detections in the 30 images) to determine which ones correspond to a living worm. Comparison at the detection level is therefore not possible, but it gives us an approximation of the neural network's ability to filter out noise. The results are as follows (Table E.1, Table E.2).

Secondly, the lifespan metrics were calculated as discussed in section 4.4. The results are as follows in Table E.3 and Fig. E.1.

In the experiment with strain N2, the traditional algorithm obtained a lower average error whereas in the experiment with strain *daf*-2, better results were obtained with the proposed method. On average, both methods displayed a similar error

## Appendix F Error analysis

This appendix shows the best and worst plate-level predictions made by the proposed method. The survival curves of the plates with the most and least error obtained are presented below. The worst performing curves are due to errors such as those shown in Fig. 8, Fig. 9b). In these plates, such cases occur more frequently and this leads to an increase in the error of the regression network. The effect of this noise is accentuated by the fact that these plates contain few worms (10-15). For this reason, we analyzed the error per condition and not per plate, in order to increase the sample size and reduce the influence of noise (Fig. F.1).
